# The Dose-Dependent Effects of Spironolactone on TGF-*β*1 Expression and the Vulnerability to Atrial Fibrillation in Spontaneously Hypertensive Rats

**DOI:** 10.1155/2021/9924381

**Published:** 2021-09-27

**Authors:** Mirong Tang, Yan Chen, Fuqing Sun, Liangliang Yan

**Affiliations:** ^1^Department of Cardiac Surgery, Fujian Medical University Union Hospital, Fuzhou City 350001, China; ^2^Department of Ultrasound,Fujian Maternity and Child Health Hospital, Affiliated Hospital of Fujian Medical University, Fuzhou City 350001, China; ^3^Department of Interventional Catheter Room, Department of Cardiovascular, Fuqing Municipal Hospital, Fuqing Municipal Hospital Affiliated to Fujian Medical University, Fuzhou City 350300, China

## Abstract

**Objective:**

This study tends to assess the dose-dependent effects of spironolactone on TGF-*β*1 expression, atrial fibrosis, and the vulnerability to atrial fibrillation in spontaneously hypertensive rats (SHRs) and tries to clarify the association of atrial fibrosis with the vulnerability to atrial fibrillation.

**Methods:**

Forty 20-week-old male SHRs were randomly divided into 4 groups (10 rats per group): 3 spironolactone groups were lower-dose group (10 mg·kg^−1^·d^−1^, dissolved in 2 ml saline solution, group SL), medium-dose group (40 mg·kg^−1^·d^−1^, dissolved in 2 ml saline solution, group SM), higher-dose group (80 mg·kg^−1^·d^−1^, dissolved in 2 ml saline solution, group SH) and one hypertension group (2 ml saline solution for stomach gavage, group H). Ten matched homologous WKY rats were set as the control group (group C). After 7 weeks of gavage, a multiple electroconductive physiological recorder was used to detect atrial electrical parameters, including P-wave duration, PR interval, and atrial effective refractory period (AERP), the inducibility, and duration of atrial fibrillation. HE staining was used to determine myocardial cell size. Masson staining was used to detect the deposition of the interstitial collagen fibers in atrial muscle. The expression of TGF-*β*1 was detected by immunohistochemistry and western blot.

**Results:**

Compared with group C, the myocardial cell size, atrial fibrosis, TGF-*β*1 expression, P-wave duration, PR interval, AERP, inducibility, and duration of atrial fibrillation in group *H* were conspicuously increased (*p* < 0.05); compared with group H, there was no significant difference in the myocardial cell size, atrial fibrosis, TGF-*β*1 expression, and electrophysiological indexes in group SH upon spironolactone intervention (*p* > 0.05); compared with group H, the myocardial cell size, atrial fibrosis, the expression of TGF-*β*1, P-wave duration, PR interval, the inducibility, and duration of atrial fibrillation in the group SL and group SM were all decreased (*p* < 0.05); compared with group SM, the effect in the group SL was more prominent (*p* < 0.01).

**Conclusion:**

Hypertension can lead to cardiomyocyte hypertrophy, deposition of interstitial fibrosis in myocardial tissue, and an increase in the vulnerability to atrial fibrillation. Spironolactone showed a certain dose-dependent effect in SHRs. Lower-dose spironolactone was superior to higher-dose spironolactone in the aspect of reducing hypertensive atrial fibrosis and TGF-*β*1 expression, as well as preventing the occurrence of atrial fibrillation.

## 1. Introduction

Hypertension often coexists with atrial fibrillation (AF); they are age-dependent and share common risk factors and pathophysiological mechanisms. Hypertension promoted AF by activating the Renin Angiotensin Aldosterone System (RAAS), which promoted left ventricular hypertrophy and left atrial remodeling [1–3]. In patients with hypertension, drug therapy can control the structural changes of the heart and prevent the occurrence of AF [4, 5]. Upstream therapies targeting to block RAAS have been emerging as a novel approach for primary prevention of AF. Spironolactone, for one, has become the research hotspot because it can reduce myocardial fibrosis. Spironolactone possesses antiandrogenic properties that will cause side effects including endocrine system and electrolyte disorders. Hence, a proper dose of spironolactone has attracted attention from many scholars, and many of them conducted intervention studies by selecting different doses of spironolactone on a variety of experimental models.

Transforming growth factor-1 (TGF-*β*1) is a cytokine that has a variety of biological effects. It is the strongest extracellular matrix deposition promoter ever discovered and is considered to be a key fibrogenic factor. On the other hand, the vulnerability to AF has become another hot spot; most researchers tend to define it as the difficulty of inducing AF when there is additional stimulation on the atrial. Most scholars tend to equate the vulnerability to AF with the inducibility of AF. In this study, we investigated the relationship between spironolactone and atrial fibrosis by using different doses of spironolactone in the intervention treatment of spontaneously hypertensive rats (SHRs) and explore the dose-dependent effects of spironolactone on TGF-*β*1 expression and the vulnerability to AF.

## 2. Materials and Methods

### 2.1. Experimental Animals and Grouping

In this study, forty 20-week-old CL male SHRs and 10 matched homologous healthy WKY rats were purchased from Shanghai SLAC Laboratory Animal Co, Ltd (Shanghai, China). The SHRs were randomly divided into 4 groups using a random number table with 10 rats per group: lower-dose spironolactone group (10 mg·kg^−1^·d^−1^, dissolved in 2 ml saline solution, group SL), medium-dose spironolactone group (40 mg·kg^−1^·d^−1^, dissolved in 2 ml saline solution, group SM), higher-dose spironolactone group (80 mg·kg^−1^·d^−1^, dissolved in 2 ml saline solution, group SH), and hypertension group (2 ml saline solution, group H). The WKY rats were taken as the control (group C). Stomach gavage was given to each rat once daily for consecutive 7 weeks. All animal experiments were carried out in accordance with both the National Animal Management Regulations and the Fundamental Guideline for Proper Conduct of Laboratory Animal of Fujian Province.

### 2.2. Devices and Reagents

Devices and reagents are as follows: Spironolactone (Minsheng Pharma., Hangzhou, China; H33020070); Softron intelligent noninvasive sphygmomanometer (Softron Biotechnology, Japan); Masson staining kit (Zhongshan Golden Bridge Biotechnology, Beijing, China); rabbit anti-mouse TGF-*β*1 antibody (Santa Cruz, SC-146, USA); multiple electroconductive physiological recorder (Huanan Medical, Henan, China).

### 2.3. Research Methods

#### 2.3.1. Measurement of Blood Pressure, Heart Rate, and Serum Potassium

After a week of adaptive feeding, blood pressure and heart rate were determined for all rats every week, and the average value of 3 repeated measurements was taken. The measurement time for each rat lasted about 30 min. Caudal vein blood was sampled for measurement of serum potassium.

#### 2.3.2. Measurement of the Left Atrial (LA) Size, Myocardial Cell Size, and Interstitial Collagen Fiber Volume Fraction

Twenty-week-old rats before drug intervention and 28-week-old rats after were required to receive echocardiography. Ten percent ketamine (3 ml/kg) was given by intraperitoneal injection for anesthesia, and then, the LA size was measured using the ultrasonic diagnostic apparatus (Vivid 7, GE), with the anterior and posterior diameter (mm) measured at the long axial section of the left ventricle. Afterward, the rats were taken for electrophysiological examination, and their atrial tissue was taken for paraffin embedding, HE staining, measurement of myocardial cell size, and Masson staining. The ratio of myocardial interstitial collagen fibrosis area to the overall area of the view was calculated.

### 2.4. Immunohistochemistry for TGF-*β*1 Protein Determination

Immunohistochemical staining for TGF-*β*1 protein determination was performed strictly per the instructions of the hypersensitive two-step method (nonbiotin) detection kit (PV-9001). Briefly, paraffin-embedded atrial tissue blocks were used for TGF-*β*1 protein determination. IPP6.0 software was applied to calculate the integrated optical density (IOD) of the positively stained areas of the view. The protein level of TGF-*β*1 in each rat was expressed as the ratio of IOD to the average area.

### 2.5. Western Blot

The expression of the TGF-*β*1 protein in rat atrial tissue was quantitated by western blot. Firstly, around 100 mg of rat atrial tissue samples was taken for exposure to lysis buffer, and proteins were extracted. Following a quantification performed using the PIERCE's BCA protein assay kit, the proteins were denatured, and then, 20 (aliquots) of them was separated on sodium dodecyl sulfate-polyacrylamide gels (SDS-PAGE) (Pulilai, B1006) and sequentially transferred onto polyvinylidene fluoride membranes (Millipore, IPVH00010, USA). After that, the membranes which carried the proteins were blocked with 5% bovine serum albumin (BSA) in Tris-buffered saline + Tween (TBS-T) overnight at 4°C. Rabbit anti-TGF-*β*1 (1 : 200, SC-146, Santa Cruz, USA) and goat anti-rabbit coupled with peroxidase (1 : 1000) were used for immunodetection. Image J 2x image analysis system was employed to calculate the grey value of each protein band. The protein expression of TGF-*β*1 was presented as grey value (target protein band)/grey value (*β*-actin protein band).

### 2.6. Electrophysiological Examination

After 7 weeks of drug intervention, all rats were treated with 10% ketamine (3 ml/kg) by intraperitoneal injection for anesthesia, and then, their limbs were fixed. Body surface electrocardiogram was connected to test P-wave duration and PR interval. Atrial effective refractory period (AERP, ms) is defined as the longest S1-S2 interval that failed to capture (output of stimulation parameters: double threshold; pulse-width: 2.5 ms) and was measured following the steps below: right jugular vein was separated and a heparinized 2F4 electrode was sent to the right atrial appendage through the jugular vein under X-ray; a multiple electroconductive physiological recorder was connected with the electrode fixed to an optimal position where the atrial wave amplitude is higher than the ventricular wave amplitude; programmed stimulation (S1S2 stimulation) was applied, with a train of 8 basic stimuli (S1S1 x8) followed by a single extrastimuli (pre-S2) at 5 ms decrements; the basic cycle lengths were set to 150 ms and 120 ms, and S2 stimulus progressively decreased 5 ms after 90 ms until atrial refractory. To induce AF, atrial burst pacing was performed (S1S1 20 ms for consecutive 30 s) and repeated 3 times. The appearance of a typical F-wave following the disappearance of a P-wave indicates the initiation of AF. The inducibility and duration of AF in each group were recorded. The duration of AF is defined as the interval between the initiation of AF and the termination of AF (output of stimulation parameters: double threshold; pulse-width: 2.5 ms).

### 2.7. Statistical Analysis

All values were expressed as mean ± standard deviation ($\bar{\chi }$ ± S) and analyzed on SPSS 20.0. All indicators were tested by normal distribution and homogeneity test for variance. Comparisons among groups were analyzed by one-way analysis of variance (ANOVA), while comparisons between two groups were tested by LSD-t-test. The Chi-square test was used to analyze the comparisons regarding the inducibility of AF between groups. *p* < 0.05 was considered to be statistically significant. Graphpad prism 5 was applied for plotting.

## 3. Results

### 3.1. Comparison of Blood Pressure, Heart Rate, Serum Potassium, and LA Size in Rats

As detailed in Table 1, the rats in group H, SH, SM, and SL had dramatically increased blood pressure in comparison with those in group C after treatment (*p* < 0.01), while the blood pressure in group H was not statistically different from that in group SH, SM, and SL (*p* > 0.05). In terms of LA size, it was remarkably increased in group H after treatment relative to that in group C (*p* < 0.01), but there was no significant difference among the spironolactone group. Additionally, no statistical difference was noted regarding the heart rate and serum potassium among these groups (*p* > 0.05).

### 3.2. Comparison of Myocardial Cell Size and Interstitial Collagen Volume Fraction

**Figure 1:**
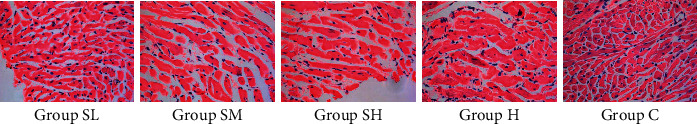
HE staining of atrial tissue.

**Figure 2:**
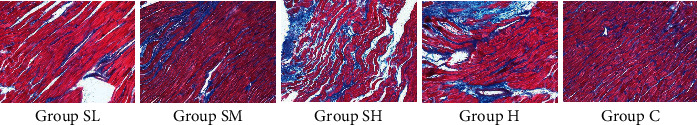
Masson staining of atrial tissue.

The detailed information is shown in Table 2 and Figures 1 and 2. Compared with group C, the myocardial cell size and interstitial collagen volume fraction in atrial tissue both were pronouncedly increased in group H (*p* < 0.01), but there was no significant difference between group H and group SH (*p* > 0.05). Besides, compared with group H, the two indicators both were decreased in group SL and group SM, and the effect was much notable in group SL (*p* < 0.05).

TGF-*β*1 expression in the atrial tissue was measured by immunohistochemical staining and western blot.

**Figure 3:**
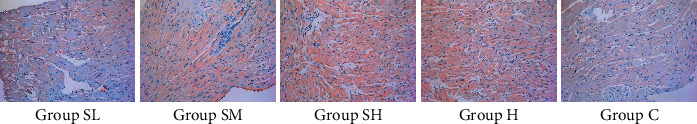
Immunohistochemical staining of TGF-*β*1 in atrial tissue.

**Figure 4:**
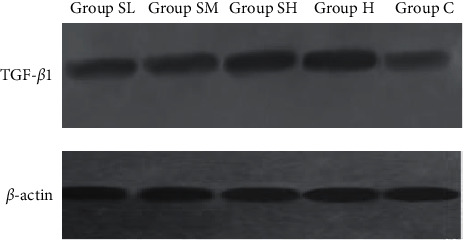
Expression of TGF-*β*1 protein by western blot.

As presented in Table 3 and Figures 3 and 4, compared with group C, the protein expression of TGF-*β*1 in atrial tissue in group H was markedly elevated (*p* < 0.01). Compared with group H, there was no significant difference with group SH (*p* > 0.05), whereas TGF-*β*1 in group SM and group SL was remarkably reduced, and the effect was much significant in group SL (*p* < 0.05).

### 3.3. Electrophysiology

**Figure 5:**
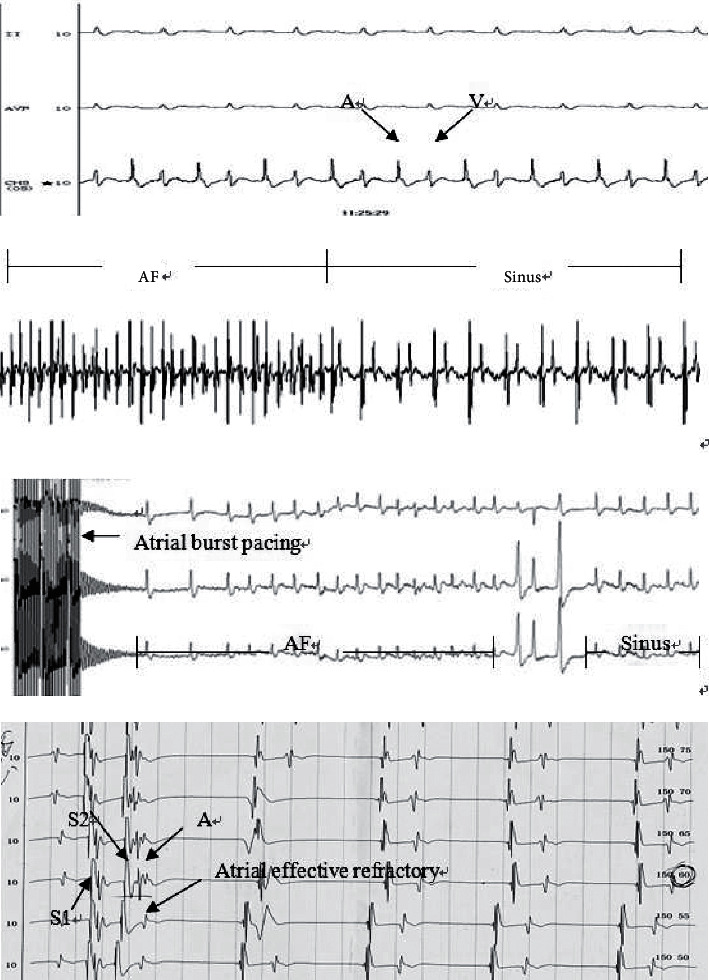
(a) Electrophysiological examination: the optimal position of atrial wave amplitude higher than ventricular wave amplitude. (b) AF turns into sinus rhythm. (c) Atrial Burst stimulation (S1S1 20 ms): continuous stimulation for 30 s was used to induce AF, which was repeated 3 times. The disappearance of the P-wave and the occurrence of a typical F-wave were the markers of AF. (d) Atrial effective refractory period (AERP): AERP is defined as the longest s1-S2 interval without atrial capture.

As shown in Table 4 and Figure 5, compared with group C, the P-wave duration, PR interval, AERP, the inducibility, and duration of AF in group H were all appreciably increased (*p* < 0.05). However, there was no significant difference in these indicators between group H and group SH (*p* > 0.05) while in group SL and group SM, the P-wave duration, PR interval, the inducibility, and duration of AF were all decreased in comparison with those in group H (*p* < 0.05); in particular, the indicators in group SL were decreased more significantly (*p* < 0.05).

## 4. Discussion

New findings from our research showed that the low-dose spironolactone can reduce myocardial hypertrophy and deposition of cardiac chamber collagen fibers and decrease susceptibility to atrial fibrillation by reducing the expression of atrial TGF-*β*1; different doses of spironolactone group had a certain dose-dependence.

Upstream therapy of atrial fibrillation has become a research hotspot in recent years, such as angiotensin-converting enzyme inhibitor (ACEI), angiotensin II receptor antagonist (ARB), spironolactone, and statins; spironolactone as one of the upstream treatment drugs has become the focus in the field of electrophysiology. Also, the aldosterone receptor antagonists can alleviate myocardial fibrosis, inhibit the excessive secretion of aldosterone, and reduce the phenomenon of “aldosterone escape” [6–8]. ACEI and ARB are popular among scholars for their appropriate therapeutic dose since they do not play an obvious role [9].

So as for spironolactone, varying doses may have different effects on atrial remodeling in SHRs. Most scholars chose 5–80 mg·kg^−1^·d^−1^ spironolactone for intervention studies in various animal models [10–12]. It was found that lower-dose spironolactone can reduce the deposition of type I collagen in the myocardium, playing an inhibitory role in myocardial fibrosis in hypertensive rats during the period of blood pressure acceleration while higher-dose spironolactone can reduce peripheral resistance and ventricular wall tension by affecting water and sodium metabolism, in turn reducing the adaptive hypertrophy of cardiomyocytes and improving ventricular remodeling. Pereira and Mandarim-de-Lacerda [13] conducted an intervention study for the effect of different doses of spironolactone (5 mg·kg^−1^·d^−1^, 10 mg·kg^−1^·d^−1^, and 30 mg·kg^−1^·d^−1^) on a 20-week-old hypertensive rat for 13 weeks in total, finding that spironolactone made an effect on blood pressure in rats in a dose-dependent manner, but there was no dose-dependent correlation with cardiac structure changes and interstitial fibrosis. The differences in the above research results may be due to varied experimental models or the dissimilar effects of different doses of spironolactone. Recently, there have been relatively few studies on the dose of spironolactone. Interestingly, we used spironolactone at 10 mg·kg^−1^·d^−1^, 40 mg·kg^−1^·d^−1^, and 80 mg·kg^−1^·d^−1^ for intervention study in SHRs.

Different from previous studies, the results of our study showed that different doses of spironolactone had little effect on blood pressure after the intervention, and its effect on improving fibrosis was independent of blood pressure. Moreover, hypertension can lead to increased atrial fibrosis and increased TGF-*β*1 expression. After intervention with different doses of spironolactone, the decrease of TGF-*β*1 was more significant in the low-dose spironolactone group. Therefore, it can be inferred that the low-dose spironolactone group can reduce atrial structural remodeling by reducing the expression of TGF-*β*1, and the low-dose spironolactone group is also significantly better than the medium-dose and high-dose spironolactone group in improving cardiomyocyte hypertrophy. This may be related to the difference in experimental animal models, the difference in blood pressure levels in rats, and the duration of intervention.

TGF-*β*1 is a cytokine with multiple biological effects. Activated TGF-*β*1 can inhibit the degradation of extracellular matrix (ECM) and increase mRNA expression and protein synthesis in ECM. TGF-*β*1 is the strongest accelerator for ECM deposition found so far and is considered to be a pivotal fibrogenic factor. Most scholars have proposed that cardiomyocyte hypertrophy and interstitial fibrosis are related to TGF-*β*1. Inhibiting the expression and secretion of TGF-*β*1 can attenuate myocardial collagen fiber deposition and atrial structural remodeling. Besides, increased atrial fibrosis can increase the heterogeneity of atrial electrical conduction; that is, structural remodeling leads to electrical remodeling [14, 15]. Moreover, it is confirmed that mitsugumin 53 (MG53) can regulate the atrial fibrosis induced by the TGF-*β*1 signaling pathway [16], while atrial fibrosis plays a critical role in AF by the TGF-*β*1/Smad pathway [17]. As such, our study found that hypertension could lead to increased atrial fibrosis and elevated TGF-*β*1 expression. After intervention with different doses of spironolactone, TGF-*β*1 was decreased more significantly in the lower-dose group. Given this, we speculate that lower-dose spironolactone leads to a decrease in TGF-*β*1 expression to reduce atrial structural remodeling. However, more research on genetic or pharmacological inhibition of TGF-*β*1 is required for upstream and downstream signaling pathways, and whether there are other factors behind the effects of spironolactone needs to be identified in the future.

Hypertension is often concomitant with increased LA pressure, leading to uneven expansion of the LA, prolonging the conduction time of impulses, increasing the heterogeneity of conduction, and aggravating the heterogeneity of AF impulses, which help the formation of internal reentry in the LA to promote and maintain the occurrence and development of AF [15]. The results of echocardiography and electrophysiology in this study also support the above view: the inner diameter of the LA of SHRs was dramatically increased, and AF was more likely to be induced and maintained after atrial burst stimulation. After 7 weeks of intervention with spironolactone, the LA size was decreased in all groups, but there was no statistical difference, which might be related to the small animals selected in this study and the short period of the experimental intervention.

Currently, most researchers tend to define atrial vulnerability in atrial fibrillation as the degree of difficulty to induce atrial fibrillation when additional stimulation acts on the atrium. However, in various domestic and foreign studies on the role of atrial vulnerability in the occurrence and development of atrial fibrillation, there is no unified definition of the concept of atrial vulnerability, and most scholars tend to equate atrial vulnerability (atrial fibrillation vulnerability) with the inducibility of atrial fibrillation. The vulnerability to AF is mainly involved in reduced atrial electrical conduction velocity, prolonged PR interval, shortened AERP, and increased dispersion. P-wave duration represents the time for depolarization of the left and right atria, which is not only related to the severity of fibrosis but also positively related to the risk of AF [18]. Prolonged PR interval means slowed atrioventricular conduction time. A study reported that prolonged PR interval will increase the risk of AF [19]. In the present study, we found that the P-wave duration and the PR interval of SHRs both were notably prolonged. After the intervention of different doses of spironolactone, there was no significant difference in the P-wave duration and the PR interval in the high-dose group, while those in the low-dose group were conspicuously shortened. The AERP reflects the excitability of the atrium. A shorter AERP is always accompanied by the easier transmission of the atrium by abnormal excitement and a high risk of arrhythmia. AERP dispersion reflects the uneven degree of excitability between the left/right atrium and pulmonary vein. The greater the dispersion, the more obvious the electrophysiological heterogeneity of myocytes in each part of the atrium, and the more likely it is for an abnormal electrical activity to form microreturns or conduction block in the local area, which is more conducive to the formation of AF [20, 21]. In this study, the right atrial appendage S1S2 stimuli were selected to measure AERP, with the basic cycle lengths set to 150 ms and 120 ms, respectively. The results found that there was no significant difference in AERP between the WKY group and the hypertension/spironolactone groups. These results are inconsistent with the previous assumption, considering the difference in the stimulation site. The structural remodeling of the left atrium was more obvious than that of the right atrium in SHRs, and electrical remodeling may also occur in the left atrium firstly. Therefore, if the stimulation site is selected for the left atrial appendage, the results may be different. In addition, the dispersion of atrial refractory period and slow conduction velocity may induce atrial fibrillation, which are electrophysiological markers of increased atrial vulnerability during atrial fibrillation; future studies may further investigate the dispersion of atrial effective refractory period. Although low-dose spironolactone cannot have a beneficial effect on AERP after the intervention, it can shorten the P-wave duration and PR interval, improve the vulnerability of the atrium of hypertensive rats, and prevent the occurrence of AF in hypertensive rats. The above views were also authenticated in this study by detecting the inducibility and the duration of AF through atrial burst stimulation.

In summary, this study found that spironolactone showed a certain dose-dependent effect. The lower-dose spironolactone was superior to the higher-dose spironolactone in reducing atrial fibrosis and the expression of TGF-*β*1, shortening the P-wave duration and the PR interval, and reducing the incidence as well as the duration of AF. This finding provides a theoretical basis for the application of spironolactone in the primary prevention of AF in patients with hypertension. At present, the role of fibrosis in cardiovascular disease has become a hot topic for scholars. Therefore, further exploration of TGF-*β*1 upstream and downstream signaling pathways along with their regulatory relationships and the study of AERP dispersion, atrial repolarization dispersion, atrial recovery time, and ion channel gene polymorphism and other atrial vulnerability are of great significance in the theoretical research of AF prevention and treatment.

### 4.1. Limitations

The occurrence and development of AF are closely related to the left atrium, while the left atrium is the first involved in hypertension due to increased left ventricular pressure. In this study, electrophysiological tests were performed on the right auricle by sending a 2F-4 electrode through the jugular vein under the X-ray line, yet the results may not fully reflect the electrophysiological changes of the left atrium. In subsequent relevant studies, we will choose the thoracotomy method to directly stimulate the left atrial auricle through the epicardium to detect the vulnerability to AF. On the other hand, the relationship in spironolactone dosage between rats and humans is not yet clear; hence, the results of this study cannot be simply applied to patients.

## Figures and Tables

**Table 1:** Comparison of blood pressure, heart rate, LA size, and serum potassium in rats (M±SD).

Group	Rat (number)	SBP (mmHg)	DBP (mmHg)	MBP (mmHg)	HR (bpm)	LA (mm)	K^+^ (mmol/L)
**20-week-old**							
Group SL	10	186.5 ± 7.3^*∗*^	107.2 ± 6.6^*∗*^	130.2 ± 5.7^*∗*^	338.1 ± 15.6	3.7 ± 0.3	4.0 ± 0.4
Group SM	10	182.6 ± 8.1^*∗*^	108.5 ± 7.1^*∗*^	132.2 ± 5.2^*∗*^	342.2 ± 13.8	3.4 ± 0.3	4.1 ± 0.3
Group SH	10	185.2 ± 5.2^*∗*^	106.2 ± 6.4^*∗*^	130.2 ± 8.4^*∗*^	333.6 ± 18.6	3.7 ± 0.2	3.9 ± 0.4
Group H	10	189.2 ± 8.3^*∗*^	108.5 ± 8.6^*∗*^	138.6 ± 5.5^*∗*^	340.1 ± 14.7	3.8 ± 0.1^#^	4.2 ± 0.2
Group C	10	125.2 ± 6.4	80.3 ± 5.2	91.4 ± 6.3	342.8 ± 16.9	3.2 ± 0.4	3.9 ± 0.5
**28-week-old**							
Group SL	10	188.2 ± 6.4^*∗*^	111.3 ± 7.6^*∗*^	132.6 ± 6.8^*∗*^	346.5 ± 12.4	5.2 ± 0.1	4.0 ± 0.5
Group SM	10	185.6 ± 5.3^*∗*^	110.2 ± 6.4^*∗*^	134.2 ± 5.7^*∗*^	348.2 ± 10.6	5.1 ± 0.9	3.9 ± 0.3
Group SH	10	186.2 ± 8.2^*∗*^	108.3 ± 6.2^*∗*^, ^*∗*^	133.2 ± 5.2^*∗*^	342.2 ± 11.5	5.0 ± 0.4	3.9 ± 0.4
Group H	10	193.6 ± 10.4^*∗*^	110.2 ± 9.4^*∗*^	140.2 ± 7.8^*∗*^	350.2 ± 9.4	5.9 ± 0.3^#^	3.0 ± 0.4
Group C	10	123.6 ± 5.6	82.2 ± 6.4	92.5 ± 5.4	341.2 ± 13.4	4.9 ± 0.1	3.9 ± 0.3

Blood pressure comparison with group C, ^*∗*^*p* < 0.01 and ^*∗*^^*∗*^*p* < 0.05; LA size comparison with group C, ^#^*p* < 0.01. SBP: systolic blood pressure, DBP: diastolic blood pressure, MBP: mean arterial pressure; HR: heart rate; LA: left atrial.

**Table 2:** Myocardial cell size and interstitial collagen volume fraction (M±SD).

Group	Rat (number)	HE staining myocardial cell size (um^2^)	Masson staining interstitial collagen volume fraction (%)
**28-week-old**			
Group SL	10	21016.25 ± 1604.41^&^	16.40 ± 1.65^&^
Group SM	10	29564.25 ± 2962.66^#^	21.30 ± 2.80^##^
Group SH	10	46453.67 ± 8480.74	23.10 ± 3.08
Group H	10	48224.75 ± 4657.69^*∗*^	23.40 ± 3.69^*∗*^
Group C	10	21783.75 ± 1730.17	15.40 ± 1.21

Compared with group C, ^*∗*^*p* < 0.01; compared with group H, ^#^*p* < 0.01 and ^##^*p* < 0.05; compared with group SM, ^&^*p* < 0.05.

**Table 3:** The expression of TGF-*β*1 in atrial tissue detected by immunohistochemistry and western blot (M±SD).

Group	Rat (number)	Immunohistochemistry TGF-*β*1 IOD/average area (%)	Western blot TGF-*β*1/*β*-actin grey value
**28-week-old**			
Group SL	10	9.1 ± 1.4^&^	0.91 ± 0.08^&^
Group SM	10	11.2 ± 1.6^##^	1.20 ± 0.06^##^
Group SH	10	13.1 ± 1.4	1.43 ± 0.05
Group H	10	15.2 ± 3.1^*∗*^	1.91 ± 0.16^*∗*^
Group C	10	8.9 ± 1.2	0.81 ± 0.06

Compared with group C, ^*∗*^*p* < 0.01; compared with group H, ^#^*p* < 0.01 and ^##^*p* < 0.05; compared with group SM, ^&^*p* < 0.05.

**Table 4:** Electrophysiological test results (M±SD) (unit: ms).

Group	Rat (number)	P-wave duration (ms)	PR interval (ms)	AERP (CL 150 ms)	AERP (CL 120 ms)	Inducibility of AF (%)	Duration of AF (s)
28-week-old							
Group SL	10	34.0 ± 2.0^&^	54.3 ± 1.6^&^	57.9 ± 2.3	57.8 ± 1.9	30 (3/10)^&^	5.1 ± 1.6^&^
Group SM	10	36.3 ± 1.8^##^	55.6 ± 2.1^##^	57.5 ± 1.9	57.4 ± 2.7	60 (6/10)^##^	6.5 ± 1.7^#^
Group SH	10	38.4 ± 1.9	57.4 ± 2.0	56.4 ± 1.7	56.8 ± 3.1	80 (8/10)	8.9 ± 2.2.
Group H	10	38.9 ± 1.3^*∗*^^*∗*^	57.2 ± 1.8^*∗*^^*∗*^	56.2 ± 1.4	56.2 ± 1.7	90 (9/10)	11.5 ± 5.8^*∗*^
Group C	10	33.2 ± 1.2	54.0 ± 1.4	57.2 ± 1.7	56.8 ± 1.8	40 (4/10)	4.7 ± 1.2

Compared with group C, ^*∗*^*p* < 0.01 and ^*∗*^*p* < 0.05; compared with group H, ^#^*p* < 0.01 and ^##^*p* < 0.05; compared with group SM, ^&^*p* < 0.05. AERP: atrial effective refractory period, CL: cycle length, AF: atrial fibrillation.

## Data Availability

The data used to support the findings of this study are included within the article. The data and materials used in the current study are available from the corresponding author on reasonable request.
